# Chronic morphine administration enhances nociceptive sensitivity and local cytokine production after incision

**DOI:** 10.1186/1744-8069-4-7

**Published:** 2008-02-22

**Authors:** DeYong Liang, Xiaoyou Shi, Yanli Qiao, Martin S Angst, David C Yeomans, J David Clark

**Affiliations:** 1 Department of Anesthesia, Stanford University School of Medicine, Stanford, CA, USA; 2 Veterans Affairs Palo Alto Healthcare System, Palo Alto, CA, USA

## Abstract

**Background -:**

The chronic use of opioids prior to surgery leads to lowered pain thresholds and exaggerated pain levels after these procedures. Several mechanisms have been proposed to explain this heightened sensitivity commonly termed opioid-induced hyperalgesia (OIH). Most of these proposed mechanisms involve plastic events in the central or peripheral nervous systems. Alterations in the abundance of peripheral mediators of nociception have not previously been explored.

**Results -:**

In these experiments mice were treated with saline (control) or ascending daily doses of morphine to generate a state of OIH followed by hind paw incision. In other experiments morphine treatment was initiated at the time of incision. Both mechanical allodynia and peri-incisional skin cytokine levels were measured. Myeloperoxidase (MPO) assays were used to determine neutrophil activity near the wounds. The cytokine production inhibitor pentoxifylline was used to determine the functional significance of the excess cytokines in previously morphine treated animals. Mice treated chronically treated with morphine prior to incision were found to have enhanced skin levels of IL-1β, IL-6, G-CSF, KC and TNFα after incision at one or more time points compared to saline pretreated controls. The time courses of individual cytokines followed different patterns. There was no discernable effect of chronic morphine treatment on wound area neutrophil infiltration. Pentoxifylline reduced cytokine levels and reversed the excess mechanical sensitization caused by chronic morphine administration prior to incision. Morphine treatment initiated at the time of incision did not lead to a generalized enhancement of cytokine production or nociceptive sensitization in excess of the levels observed after incision alone.

**Conclusion -:**

The enhanced level of nociceptive sensitization seen after incision in animals chronically exposed to morphine is associated with elevated levels of several cytokines previously reported to be relevant to this incisional pain model. The cytokines may be functional in supporting nociceptive sensitization because pentoxifylline reverses both peri-incisional skin cytokine levels and OIH. Opioid administration beginning at the time of incision does not seem to have the same cytokine enhancing effect. Approaches to postoperative pain control involving a reduction of cytokines may be an effective way to control excessive pain in patients chronically using opioids prior to surgical procedures.

## Introduction

While opioids are a mainstay of treatment for chronic pain and are second only to NSAIDS terms of the number of prescriptions written for common chronic pain syndromes [1], problems with long term use are becoming better recognized. Tolerance and dependence on these drugs have been studied for some time. More recently, the phenomenon of opioid-induced hyperalgesia (OIH) has come under study (see ([2-6]) for recent reviews). This form of hyperalgesia has been noted in human populations maintained long term with methadone [7-11], treated with morphine for back pain [12], and infused with the potent short-acting opioid remifentanil in experimental pain laboratories [13-18]. Based largely on studies in laboratory animals, several mechanisms have been suggested including sensitization of peripheral nociceptors [19-21], enhanced production and central release of excitatory amino acid (EAA) and peptide neurotransmitters [22,23], diminished uptake of EAA neurotransmitters in the spinal cord [24], and enhanced descending facilitation of spinal nociceptive neurotransmission from the rostral ventromedial medulla (RVM) [25]. Many of these responses represent mechanistic counterbalances to the acute effects of opioid receptor activation.

Clinically, post-incisional pain has been observed to be more severe in chronically opioid consuming patients [26]. These patients consume several times the normal doses of opioids in the perioperative period and do not always achieve the same level of comfort as previously opioid naïve patients [26,27]. The situation is sufficiently widely recognized that guidelines for the management of these patients have been provided [28]. Several groups have studied the phenomenon of opioid-exaggerated post-incisional pain in laboratory models. Our group has studied the problem in a rat model of incisional pain in which the animals were treated chronically with morphine prior to the incisional being made [29]. In this model it was observed that opioid treated animals displayed mechanical allodynia in excess of saline treated ones with the difference not appearing until 2 days after incision, reaching maximum at 3 days and persisting for at least 7 days. Celerier et al. observed that the administration of high dose opioids at the time of incision generated hind paw peri-incisional sensitization in excess of what was observed when using inhalational anesthesia alone [30]. Again, the opioid-induced excess hyperalgesia was prominent for up to 7 days following incision. The mechanisms responsible for the opioid-induced excess in nociceptive sensitization have not been fully explored.

Recently we observed that the acute systemic administration of opioids like morphine can substantially reduce the abundance of specific cytokines accumulating in the peri-incisional tissue [31]. Several of these cytokines including interleukin-1β (IL-1β), IL-6, tumor necrosis factor-α (TNFα) have been observed to support enhanced nociceptive sensitivity in various rodent models [32-34]. Both keratinocytes and infiltrating neutrophils were observed to be producing these cytokines near the wound edges [31]. Because it appears that the chronic effect of opioids is sometimes to cause effects paradoxically opposite to the acute effects of these drugs, we hypothesized that mice chronically treated with opioids would display exaggerated levels of skin cytokine production after hind paw incisions. This would explain some portion of the exaggerated nociceptive sensitization seen in these animals. Such observations would be novel and might suggest approaches to better controlling pain in vulnerable clinical populations.

## Results

### 3.1 Effects of chronic morphine treatment on post-incisional mechanical sensitization

**Figure 1:**
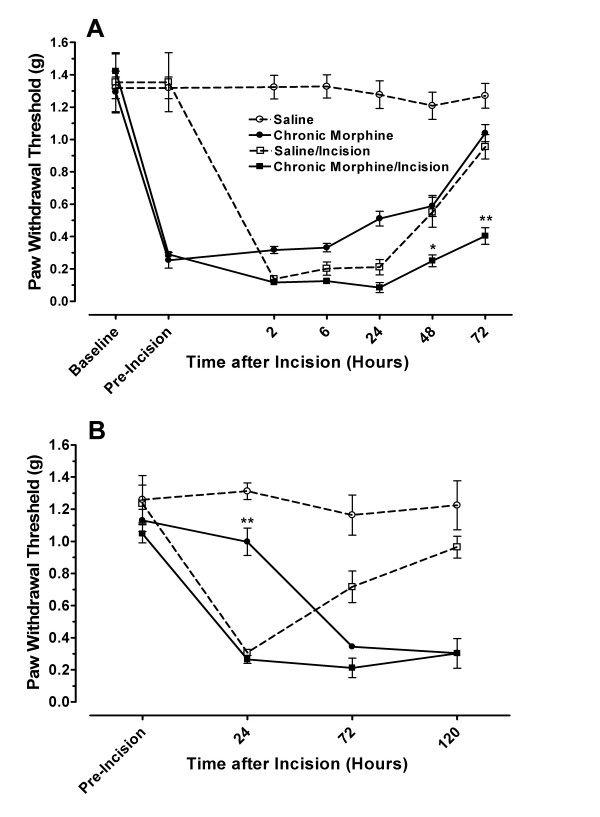
**Mechanical nociceptive thresholds after drug pretreatment and hindpaw incision.** Panel A displays the mechanical thresholds of mice in four different treatment groups: saline pretreatment/no incision, saline pretreatment/incision, morphine pretreatment/no incision, morphine pretreatment/incision. The values labeled pre-incision represent the nociceptive thresholds measured after 4 days of saline or morphine treatment but prior to hind paw incision. In panel B data are presented representing mechanical nociceptive thresholds in mice undergoing saline or morphine treatment beginning at the time of incision. Nociceptive thresholds were measured immediately before that day's dose of morphine. The statistical analysis presented reflects the results of two-way ANOVA comparing saline/incision values to morphine/incision ones. *p < 0.05, **p < 0.01, N = 6/group.

The mechanical nociceptive thresholds for mice at baseline and after 4 days of morphine (chronic morphine) or saline treatment were first measured. In Figure 1A it can be seen that prior to incision morphine treated mice developed mechanical allodynia while saline treated mice had no change in withdrawal thresholds. If no hindpaw incisions were made, morphine pre-treated mice recovered from this allodynia to achieve near baseline withdrawal thresholds over the following 72 hours. Other data in the figure demonstrate that incision, as expected, caused a profound but transient decrease in nociceptive thresholds. While these mice also recovered from the allodynia, mice pretreated with morphine then incised had a slower course of recovery. Specifically, the morphine pretreated/incised mice had lower mechanical withdrawal thresholds than the saline/incised mice at least to 72 hours post-incision.

Additional experiments were completed in which chronic daily saline or morphine treatment was initiated at the time of incision and continued for 4 days according to our established ascending dose protocol. This protocol was designed to be similar to the titration of opioids which might occur post-tissue injury. At the 72 and 120 hour time points after incision, nociceptive thresholds for the morphine and morphine/incision treatment groups were not statistically different (Figure 1B). Previous experimentation established that the 120 hour time point was the time point of maximum morphine-induced sensitization in this model [35]. Thus we did not detect an additive nociception enhancing effect of chronic morphine with incision using this paradigm.

### 3.2 Effects of chronic morphine treatment on post-incisional cytokine generation

**Figure 2:**
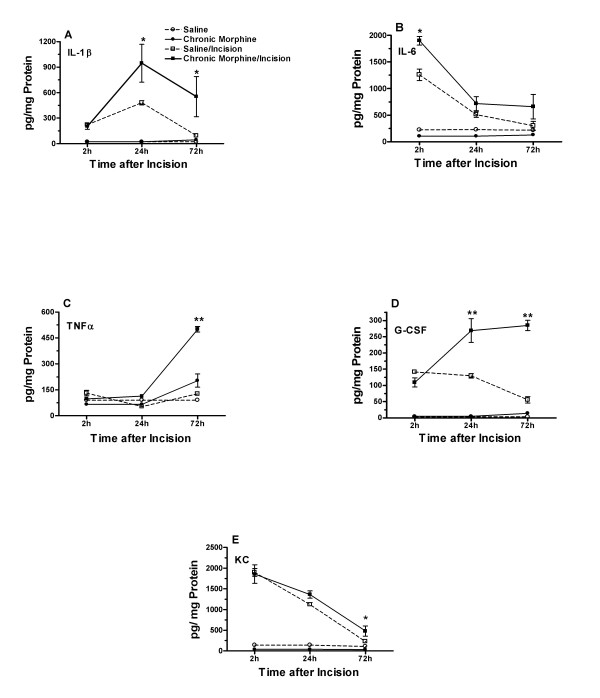
**Peri-incisional skin cytokine levels after saline or morphine pretreatment.** The figure displays the mechanical thresholds of mice in four different treatment groups: saline pretreatment/no incision, saline pretreatment/incision, morphine pretreatment/no incision, morphine pretreatment/incision. The cytokine levels were assessed at time points out to 72 hours after incision based on the results of the experiments presented in Figure 1. The statistical analysis presented reflects the results of two-way ANOVA comparing saline/incision values to morphine/incision ones over the 2 to 72 hour time period. *p < 0.05, **p < 0.01, N = 8/group.

In other groups of mice, the skin levels of 5 cytokines were measured using the chronic morphine prior to incision paradigm. Those data are presented in Figure 2. In the absence of incision, the treatment of mice with morphine for 4 days did not alter the baseline skin levels of any of these cytokines compared with saline pretreated animals.

Hind paw incision did lead to increases in the skin levels of all 5 cytokines, though the magnitude of the changes and the time course of expression was particular to each mediator. The expression patterns observed for these 5 mediators were similar to those previously reported by our laboratory after incision for non-opioid treated mice [31,36]. For all of the tested cytokines, IL-1β, IL-6, TNFα, G-CSF and KC, chronic morphine treatment prior to incision lead to an enhanced tissue level of cytokine compared with levels in saline treated then incised mice at one or more time points. Only for the cytokine, IL-6, was there no significant difference between the saline and morphine pretreated incised groups 72 hours after incision, the point at which the behavioral differences were maximal (Figure 1). The magnitude of the cytokine increase after incision was up to 5-fold larger in morphine treated than saline treated groups (G-CSF, 72 hours after incision).

**Figure 3:**
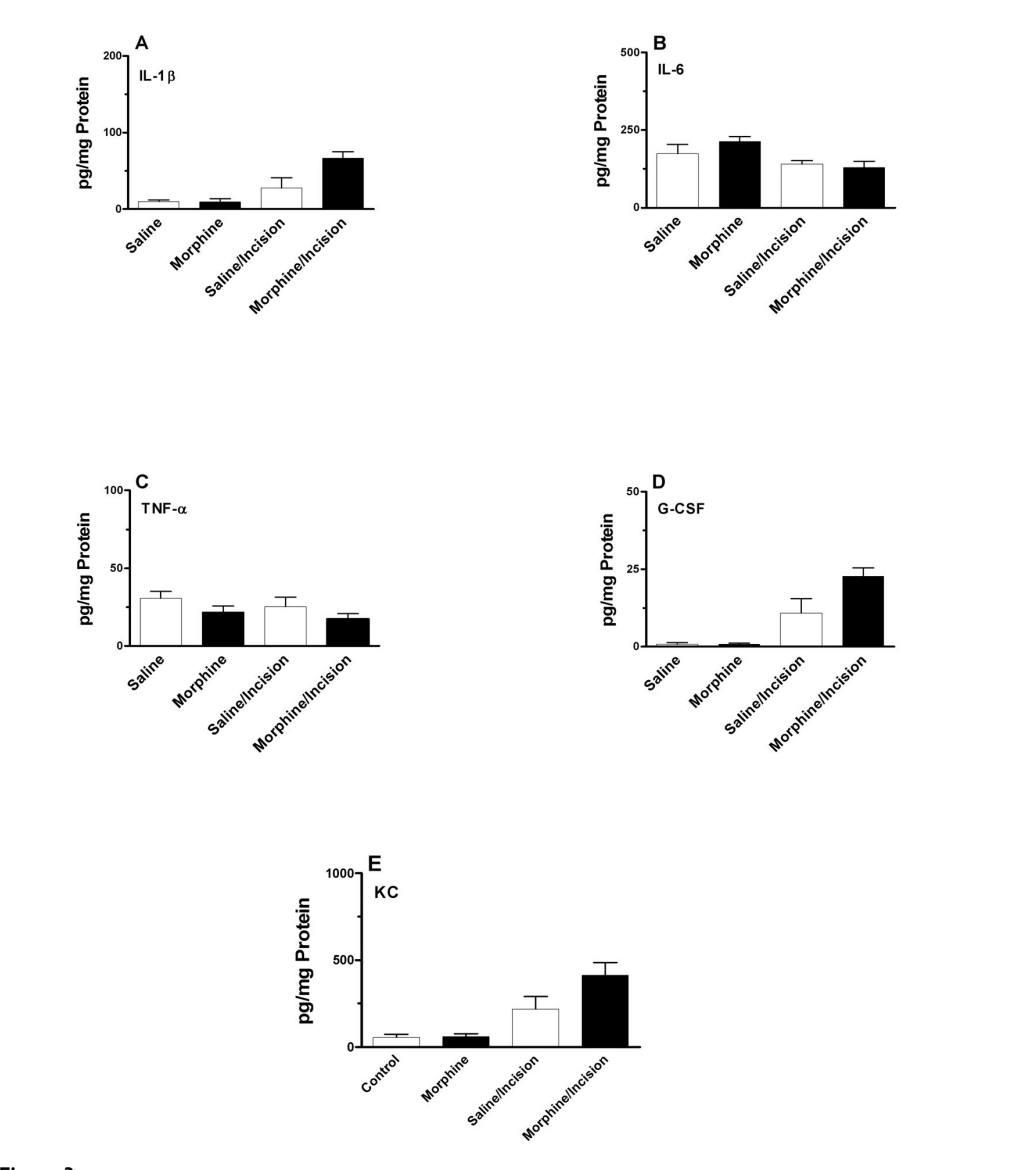
**Peri-incisional skin cytokine levels for mice treated with saline or morphine beginning at the time of incision.** The figure displays cytokine measurements from mice in four different treatment groups: saline, incision/saline, no incision/morphine, incision/morphine. The cytokine levels were assessed at 120 hours after the incisions were made (18 hours after the last dose of morphine). Statistical analysis was performed to detect differences between each set of conditions. N = 8/group.

Additional experiments measuring skin cytokine levels from mice undergoing morphine treatment beginning at the time of incision failed to demonstrate any significant alterations in these levels when compared saline, morphine or saline/incision conditions (Figure 3). Additionally, the overall cytokine levels in the skin surrounding the wounds was much lower than observed in the experiments presented in Figure 2.

### 3.3 Effects of chronic morphine treatment on post-incisional MPO activity

**Figure 4:**
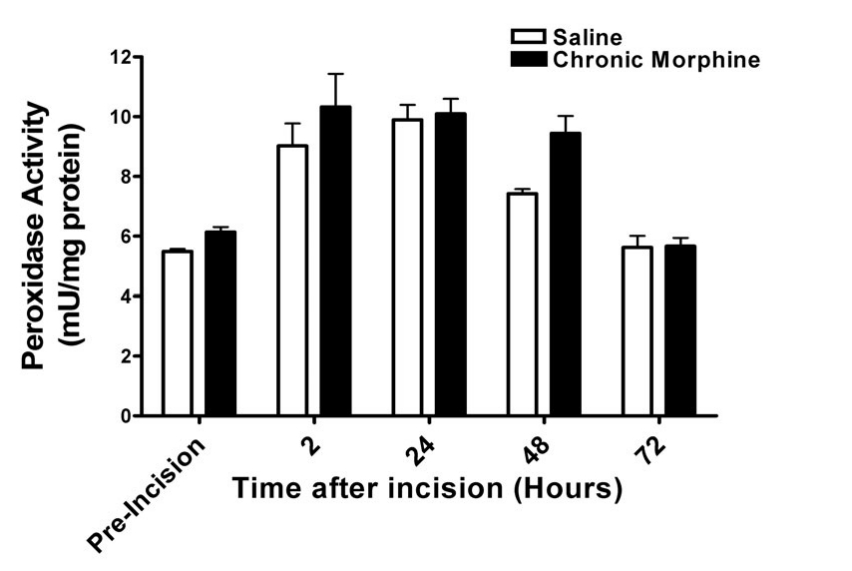
**Myeloperoxidase (MPO) activity after hind paw incisions.** For these experiments MPO activity was measured in peri-incisional skin as an index of infiltrating neutrophil activity. Skin was harvested from separate groups of mice at the indicated time points and processed for MPO assays as described in Methods. The time course for analysis was the same as that used for the behavioral and cytokine assays. Statistical analysis failed to detect between group differences at any of these time points. N = 8/group.

Because infiltrating neutrophils are a potential source of cytokines in incisional wounds and because acute morphine administration can alter neutrophil infiltration [31], we sought to determine whether the morphine-related differences in cytokine levels were attributable to differences in the peri-incisional levels of these infiltrating cells. We chose for these measurements the myeloperoxidase assay (MPO) which has excellent correlation with immunohistochemical neutrophil counts in these mouse hind paw incisions [31]. In Figure 4 data are presented showing that incising the hind paw does lead to a rapid but transient rise in neutrophil skin abundance. However, chronic morphine pretreatment did not significantly alter the degree of infiltration when compared with saline pretreated mice. Specifically, MPO levels were not higher in the skin of morphine treated then incised skin when compared with saline treated then incised skin at the 2, 24 or 72 hour time points when the levels of specific cytokines were enhanced (Figure 2).

### 3.4 Pentoxifylline effects on morphine enhanced post-incision allodynia and cytokine generation

**Figure 5:**
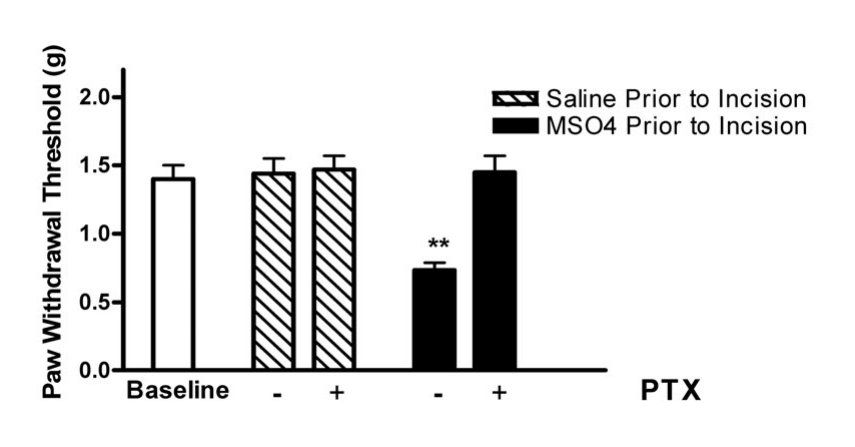
**Mechanical allodynia after incision as affected by morphine and pentoxifylline.** In these experiments mice were subjected to nociceptive testing of the hind paw to establish the baseline threshold. Mice were then pre-treated with either saline or morphine for 4 days prior to hind paw incisions being made. After incision, groups of mice were treated daily with daily intraperitoneal saline or pentoxifylline as described in Methods. Nociceptive testing 72 hours after hind paw incision (2 hours after the last dose of pentoxifylline). **p < 0.01, N = 8/group.

While we had observed to this point a correlation between incision area cytokine levels and the enhancement in post-incision allodynia caused by chronic morphine administration, we had no functional evidence for the participation of these substances. We therefore administered the broad spectrum cytokine production inhibitor pentoxifylline on a daily basis after incisions were made. The pentoxifylline dose (50 mg/kg) and daily injection schedule chosen for these experiments has been shown by others to reduce nociceptive sensitization in mouse and rat models of neuropathic pain [37,38]. In Figure 5 data are presented demonstrating that pentoxifylline administered daily after the time of hind paw incision completely eliminated the morphine-enhancement of nociception normally observed at 72 hours. There was no effect on the mechanical nociceptive thresholds of mice that underwent hind paw incision after chronic saline pretreatment. In additional experiments we observed that pentoxifylline reduced skin cytokine levels 72 hours after incision in morphine pretreated mice. The levels were reduced by 74%, 55% and 53% for G-CSF, IL-1β and KC respectively (p < 0.05 vs. vehicle-treated mice). For IL-6 and TNFα, pentoxifylline failed to significantly reduce cytokine levels.

## Discussion

Opioid-induced hyperalgesia (OIH) is a state of paradoxically enhanced pain sensitivity observed in both humans and animals after chronic exposure to opioids including morphine, oxycodone, fentanyl, heroin and others. To-date most explorations of this phenomenon's mechanism have focused on alterations in functional elements within the central nervous system and to a lesser degree neuroplastic changes involving primary afferent sensory neurons [2]. Our studies were focused further in the periphery and involved opioid effects on skin cytokine levels both before and after hindpaw incision. Peripheral cytokine production had not prior to this time been implicated in the mechanism of OIH. Our results using the C57BL/6 strain of mice reproduced those we had previously reported for rats in that morphine treatment both lead to enhanced nociceptive sensitivity in unperturbed animals, and significantly enhanced the sensitization of hindpaws for several days after incision [29]. More importantly, we went on in the present studies to show that the peri-incisional expression of cytokines previously demonstrated to be inhibited by morphine given acutely before incision [31] were moderately to strongly enhanced in the tissues of mice chronically pretreated with morphine. These cytokines included IL-1β, IL-6, TNFα, G-CSF and KC. Of these, only IL-6 was not elevated in the previously morphine treated mice compared to saline treated mice 72 hours after incision, the time point at which the difference in mechanical sensitization was maximal. The broad spectrum cytokine inhibitor pentoxifylline reduced both the excess cytokines and excess sensitization measured in the morphine treated animals. The specific site of action of pentoxifylline was not determined, though pentoxifylline has been noted to reduce cytokine production by keratinocytes in culture [39] as well as after ultraviolet light burns and psoriatic skin [40,41]. Finally, we demonstrated that the initiation of morphine treatment at the time of incision did sensitize the hind paws of mice. However, there did not appear to be any interaction of morphine administration with nociceptive sensitization in this context. Furthermore, morphine administered under these circumstances did not lead to any significant differences in wound area cytokine levels. Taken as a whole, our data suggest that chronic morphine treatment prior to incision leads to excess production of cytokines thus enhancing nociceptive sensitization after incision. Administration of morphine beginning at the time of incision does sensitize mice, but this is not in excess of what is seen with morphine treatment of incision naïve mice, and there is little enhancement of cytokine production. Thus cytokine mediation of exaggerated post-incisional nociception is more relevant to the treatment of chronically opioid consuming patients than otherwise opioid naïve patients being treated for postoperative pain.

A range of mechanisms causing OIH have been proposed and recently reviewed [2]. The principal mechanisms currently considered responsible for OIH include those leading to enhanced function or activity of afferent fibers, second order or projection neurons and descending facilitory fibers from the brainstem. For example, chronic morphine treatment leads to the up-regulation of production and release of neurotransmitters by primary afferent nerve fibers [42,43]. Once released, glutamate reuptake systems in the spinal cord function less efficiently by virtue of lower levels of expression of neuronal and glial glutamate reuptake transporters [24]. Also, it appears that for a given level of primary neurotransmitter like glutamate or substance P that more nociception related behavior is generated in mice having been treated for several days with morphine. Finally, descending facilitation from the RVM is enhanced in chronically morphine treated animals constituting an independent pathway for facilitation of nociceptive signal transmission [25]. In our own previous studies we had implicated beta-2 adrenergic receptors located on peripheral nerve terminals as being linked to OIH [21]. It should be recognized that one key feature separating our observations of cytokine participation in post-incisional OIH versus most other observations is that this peripheral cytokine mechanism only seems to be operative when tissue has been damaged. We failed to find morphine-induced differences in skin cytokine levels prior to incision and thus cannot invoke these mediators as supporting hyperalgesia observed in uninjured mice. This pattern of expression distinguishes our observations from those of others who found upregulation of IL-1β, IL-6 and TNFα in spinal cord microglia after morphine treatment even without additional trauma [44,45].

Our previous studies used immunohistochemical techniques to demonstrate that both infiltrating neutrophils and peri-incisional keratinocytes produce the cytokines we measured as elevated after incision [31]. However, as was the case in our previous experiments using acute morphine administration, the degree of peri-incisional neutrophil infiltration did not correlate with peri-incisional cytokine levels. Importantly, chronic morphine treatment was not seen to alter wound area neutrophil infiltration. Thus a simple lessening of neutrophil migration does not seem to be the explanation for the changes in inflammatory mediators observed in our experiments. The function of the infiltrating cells was not, however, directly assessed. Systemic opioid administration has been noted to alter immune system functioning in a number of ways [46].

Future studies addressing this issue will need to address at least two possible mechanisms. The first mechanism involves the participation of peripheral afferent neurons in controlling keratinocyte cytokine production. The second focuses on opioid actions on the keratinocytes themselves. Addressing neural mechanism first, chronic opioid exposure up-regulates the production of SP and CGRP at the level of both mRNA and protein [22,42,43]. Though commonly thought of participating only in augmenting nociceptive signal transmission, these neuropeptides act on keratinocytes to enhance the production of IL-1β, IL-6, TNFα and other cytokines [47]. Thus the enhanced availability and release of these neuropeptides after incision might explain the augmented levels of cytokines in the incisional wounds of morphine treated mice. Second, opioid receptors are found on keratinocytes, and keratinocytes produce opioid peptides [48,49]. These receptors have clear roles in modulating keratinocyte differentiation, wound healing, and inflammatory responses [50]. While little investigation has focused directly on opioid modulation of keratinocyte cytokine production, it could be hypothesized that chronic exposure of keratinocytes to opioids could alter their cytokine producing capacity thus leading to enhanced cytokine levels after incision. This possibility could be addressed directly.

Our studies suggest that some of the cytokines analyzed may be of special functional interest. The cytokines most likely to be involved in supporting OIH in our incisional model include IL-1β, G-CSF and KC. For all three of these cytokines peri-incisional skin levels were augmented in morphine treated mice after incision to levels above those observed in the opioid naïve animals. Furthermore, the cytokine production inhibitor pentoxifylline reduced the skin concentration of all three of these cytokines by >50% in the morphine treated animals three days after incision at the same time as pentoxifylline reversed the excess sensitization of the hind paws. A variable degree of information is available from the literature to assist in interpreting these observations. With respect to IL-1β, detailed work has been provided showing that sub-picogram quantities of subcutaneous IL-1β cause mechanical allodynia. This allodynia is probably mediated by activation of central metabotropic glutamate receptors [51,52]. Thus, excess levels of this cytokine might reasonably be expected to enhance nociception. For KC, there appears to be little information concerning the direct effects of KC on nociceptive thresholds. On the other hand, investigators have linked this cytokine to the recruitment of opioid peptide expressing neutrophils in inflamed skin. Blockade of KC activity reduced this peripherally mediated anti-nociception [53]. However, given the lack of effect of chronic morphine treatment on neutrophil recruitment in the incisional pain model, it is unclear if the previously reported KC mediated chemoattractant effects are important to our observations. Lastly, there is little work describing possible effects of G-CSF on nociceptive thresholds in control animals or in models of tissue injury. Of note, however, is that the subcutaneous administration of G-CSF in humans can cause a psoriasis-like inflammatory dermatitis. Analysis of G-CSF induced lesions revealed evidence of inflammation and elevated levels of cytokines including IL-8, IL-12 and TNFα [54]. Though we followed levels of several mediators, it is possible that additional cytokine mediators participate in the chronic morphine administration post-incision exacerbation of sensitization as only a small subset of all known cytokines were measured in our studies.

Excessive and difficult to treat pain after surgery has been noted for patients chronically consuming opioids. For example, de-Leon Casasola demonstrated in a series of clinical investigations that chronic opioid consuming patients require approximately three times greater doses of epidural opioids to control pain after surgical procedures, and that these patients tend to have more serious pain for a prolonged period after their operations [27,55,56]. Looking at required doses of parenteral morphine, Rapp et al. came to many of the same conclusions, namely that about three times as much opioid was required to control pain in chronic opioid consuming patients [26]. Importantly, it was also observed that opioid side effects were greater and the degree of pain relief was poorer despite the enhanced doses. More recently it was shown that chronic back pain sufferers develop measurable hyperalgesia after only one month of opioid administration [12]. Reviews are available further characterizing the problem and making suggestions for postoperative management of pain in chronically opioid consuming patients [28,57]. However, very little work has been done specifically directed at evaluating analgesic techniques of potential benefit to the chronic opioid consuming patient.

## Conclusion

Along with exacerbated post-incisional nociception comes excess cytokine production in animals administered morphine on a chronic basis. The excess incisional area cytokine production may explain the enhanced nociception in laboratory animals and by extension may explain some amount of the excess surgical pain suffered by humans chronically consuming opioids. This work provides a rational basis on which to design therapies aimed at reducing OIH as it impacts postoperative pain. Our work suggests that strategies designed to reduce the production of cytokines globally or perhaps reduce the production of several specific cytokines may be of benefit to opioid consuming patients. Our results to-date do not shed light on the required opioid doses or treatment durations which might be most closely associated with excess post-incisional cytokine production. In addition, different opioids such as morphine and buprenorphine have differing abilities to alter other aspects of immune function. Thus different opioids might have differing intrinsic abilities to control wound area inflammatory mediators [46]. As enthusiasm for the use of opioids for the treatment of chronic pain grows, the number of patients using opioids prior to surgical procedures is likely to rise. Therefore, the need for better postoperative pain management techniques applicable to this population is of rapidly increasing importance.

## Methods

### Animal use

All experimental protocols were reviewed and approved by Veterans Affairs Palo Alto Healthcare System institutional animal care and use committee prior to the initiation of work. Male mice 12–14 weeks old of the C57Bl/6J strain were kept under standard conditions with a 12 h light/dark cycle, an ambient temperature of 22±1°C and allowed food and water ad libitum. Mice were obtained from Jackson Laboratories (Bar Harbor, MA) and were kept in our animal facility a minimum of 1 week prior to use in experiments.

### Hind paw incision

The hind paw incision model was used as modified for mice [58]. We have used this model previously in order to study cytokine levels following incision [31,36]. Briefly, mice were anesthetized using isoflurane 2–3% delivered through a nose cone. After sterile preparation with alcohol, a 0.5 cm longitudinal incision was made with a #15 scalpel on the plantar surface of one hind paw. This incision was sufficiently deep to divide the plantaris muscle longitudinally. After briefly holding pressure to stop any active bleeding, a single 6-0 nylon suture was placed and antibiotic ointment applied. Mice were then returned to their cages on soft bedding. Nociceptive testing and tissue harvest took place at time points up to 120 hours after incision.

### Drug administration

For some groups of mice, morphine or saline vehicle was administered prior to incision. Mice received either saline injections twice per day or morphine on an ascending schedule: Day 1, 10 mg/kg twice per day; Days 2–3, 20 mg/kg twice per day; Day 4, 40 mg/kg twice per day. To accomplish this, 100 μl 0.9% NaCl with or without morphine (Sigma Chemical, St. Louis, MO) was injected subcutaneously into the skin of the back at the required times. Incision and nociceptive testing procedures began approximately 18 hours after the final dose of morphine or saline. This morphine administration protocol has been used extensively by the lab in studying opioid tolerance, dependence and hyperalgesia [21,59,60]. Alternatively, some groups of mice received the same saline or ascending morphine treatment beginning with the first injection at the time of hind paw incision or sham incision surgery.

In additional experiments pentoxifylline (Sigma) was administered after chronic pre-incisional saline or morphine treatments followed by hind paw incision. This drug was dissolved in 0.9% NaCl and injected intraperitoneally to mice at the time of hind paw incision and 24, 48 and 70 hours after the incisions were made. The dose used was 50 mg/kg.

### Nociceptive testing

Mechanical allodynia was assayed using nylon von Frey filaments according to the "up-down" algorithm described by Chaplan et al. [61] as we have used previously to detect allodynia in mice [36,62,63]. In these experiments mice were placed on wire mesh platforms in clear cylindrical plastic enclosures 10 cm in diameter and 40 cm in height. After 15 minutes of acclimation, fibers of sequentially increasing stiffness were applied 1 mm lateral to the central wound edge, pressed upward to cause a slight bend in the fiber and left in place 5 sec. Withdrawal of the hind paw from the fiber was scored as a response. When no response was obtained the next stiffest fiber in the series was applied to the same paw; if a response was obtained a less stiff fiber was applied. Testing proceeded in this manner until 4 fibers had been applied after the first one causing a withdrawal response allowing the estimation of the mechanical withdrawal threshold [64]. This data fitting algorithm allowed the use of parametric statistics for analysis. This assay is sufficiently sensitive to detect mechanical thresholds as low as 0.02 g [21].

### Cytokine analysis

To obtain skin samples for cytokine quantification animals were first sacrificed by CO2 asphyxiation and an ovular patch of full-thickness skin providing 1.5 mm margins surrounding the hind paw incisions was collected by sharp dissection. Patches from each hind paw contained approximately 12 mg tissue. These samples were placed immediately into ice cold 0.9% NaCl containing a cocktail of protease inhibitors (Complete™, Roche Applied Science, Indianapolis, IN). Approximately 750 μl inhibitor containing saline was used per 25 mg tissue. The samples were homogenized using a Polytron device (Brinkman Instruments Inc., Westbury, NY), then centrifuged for 10 min at 12,000 times gravity at 4°C. Supernatant fractions were kept frozen at -80°C until use. An aliquot was subjected to protein assay (DC Protein Assay, Bio-Rad Laboratories, Hercules, CA).

For the cytokine assays, custom Bio-Rad (Bio-Rad laboratories, Hercules, CA) Bio-Plex cytokine analysis kits were used in conjunction with the Bio-Plex system array reader according to the manufacturer's directions as described previously [36]. The specific cytokines were chosen based on our previously reported results and included IL-1β, IL-6, G-CSF, KC and TNFα [31,36]. Samples were diluted 1:2 prior to analysis in the buffer supplied, and all samples were run in duplicate or triplicate in each assay. We demonstrated previously that the dynamic range of sensitivity of this assay was sufficient to measure both baseline and incision-stimulated levels of the chosen cytokines [36]. Standard curves for each of the analyzed substances were included in each run, and sample concentrations were calculated using Bio-Plex Manager software.

### Myeloperoxidase (MPO) assay

MPO activity was measured as a biochemical index of neutrophil recruitment in the wound edge samples, and showed excellent correlation with immunohistochemical neutrophil counts in previous studies [31]. Excised skin samples were washed in PBS and homogenized in 1 ml 50 mM potassium phosphate-buffer solution with 0.5% hexadecyl trimethyl ammonium bromide (Sigma Chemical Co., St. Louis, MO) and 5 mM EDTA. The samples were then homogenized as above and centrifuged at 12,000 rpm at 4°C. MPO activities in the supernatants were then assayed using a peroxidase assay kit (Anaspec, San Jose, CA) along with supplied standards according to the manufacturer's instructions. The data were expressed as MPO activity in mU per mg protein in the supernatant samples.

### Statistical analysis

Analysis of repeated parametric measures was accomplished using a one-way ANOVA analysis of variance followed by post-hoc Dunnett's testing or a two-way ANOVA followed by Bonferroni testing. A value of p < 0.05 was taken to be significant. All data are presented as means +/- S.E.M. unless otherwise noted.

## Abbreviations

ANOVA, Analysis of Variance; CGRP, Calcitonin Gene Related Peptide; G-CSF, Granulocyte Colony; Stimulating Factor; IL, Interleukin; KC, Keratinocyte Derived Cytokine; MPO, Myeloperoxidase; NSAIDs, Non-Steroidal Anti-Inflammatory Drugs; OIH, Opioid-Induced Hyperalgesia; RVM, Rostral Ventromedial Medulla; S.E.M., Standard Error of the Mean; SP, Substance P; TNFα, Tumor Necrosis Factor Alpha.

## Competing interests

The author(s) declare that they have no competing interests.

## Authors' contributions

DL Completed primary incisional experiments with and without opioid treatments. XS Processed skin samples for myeloperoxidase and cytokine experiments. YQ Performed all cytokine analysis. DCY Supervised the analysis of all cytokine experiments and maintained cytokine analysis core. MSA Participated in the analysis and interpretation of experiments. JDC Had primary responsibility for design of experiments and organization of project.
